# Monitoring Procalcitonin in Febrile Neutropenia: What Is Its Utility for Initial Diagnosis of Infection and Reassessment in Persistent Fever?

**DOI:** 10.1371/journal.pone.0018886

**Published:** 2011-04-25

**Authors:** James Owen Robinson, Frédéric Lamoth, Frank Bally, Marlies Knaup, Thierry Calandra, Oscar Marchetti

**Affiliations:** 1 Infectious Diseases Service, Department of Medicine, Centre Hospitalier Universitaire Vaudois (CHUV) and University of Lausanne (UNIL), Lausanne, Switzerland; 2 Department of Microbiology and Infectious Diseases, Royal Perth Hospital, Perth, Australia; University of California Los Angeles, United States of America

## Abstract

**Background:**

Management of febrile neutropenic episodes (FE) is challenged by lacking microbiological and clinical documentation of infection. We aimed at evaluating the utility of monitoring blood procalcitonin (PCT) in FE for initial diagnosis of infection and reassessment in persistent fever.

**Methods:**

PCT kinetics was prospectively monitored in 194 consecutive FE (1771 blood samples): 65 microbiologically documented infections (MDI, 33.5%; 49 due to non-coagulase-negative staphylococci, non-CNS), 68 clinically documented infections (CDI, 35%; 39 deep-seated), and 61 fever of unexplained origin (FUO, 31.5%).

**Results:**

At fever onset median PCT was 190 pg/mL (range 30–26'800), without significant difference among MDI, CDI and FUO. PCT peak occurred on day 2 after onset of fever: non-CNS-MDI/deep-seated-CDI (656, 80–86350) vs. FUO (205, 33–771; p<0.001). PCT >500 pg/mL distinguished non-CNS-MDI/deep-seated-CDI from FUO with 56% sensitivity and 90% specificity. PCT was >500 pg/ml in only 10% of FUO (688, 570–771). A PCT peak >500 pg/mL (1196, 524–11950) occurred beyond 3 days of persistent fever in 17/21 (81%) invasive fungal diseases (IFD). This late PCT peak identified IFD with 81% sensitivity and 57% specificity and preceded diagnosis according to EORTC-MSG criteria in 41% of cases. In IFD responding to therapy, median days to PCT <500 pg/mL and defervescence were 5 (1–23) vs. 10 (3–22; p = 0.026), respectively.

**Conclusion:**

While procalcitonin is not useful for diagnosis of infection at onset of neutropenic fever, it may help to distinguish a minority of potentially severe infections among FUOs on day 2 after onset of fever. In persistent fever monitoring procalcitonin contributes to early diagnosis and follow-up of invasive mycoses.

## Introduction

Febrile neutropenia is a frequent complication in patients with hematological malignancies requiring prompt empirical broad-spectrum antibacterial therapy. Cultures and clinical examination fail to detect a pathogen and/or an infectious focus in 30–60% of episodes, which are classified as fever of unknown origin (FUO) [1]. As infectious and non-infectious etiologies of FUO cannot be differentiated, all patients receive maybe unnecessarily prolonged empirical antibacterial therapy. Persistence of fever despite ongoing antibiotics may suggest uncontrolled and potentially life-threatening infection due to resistant bacteria or fungi. Unresolved fever represents a major challenge for diagnosis and therapy, often resulting in multiple investigations and empirical modifications of antibacterial and/or addition of antifungal therapy [1]. New diagnostic tools are thus needed for guiding management of febrile neutropenic patients when initial microbiological and clinical documentation is lacking and in persistent fever, when resistant bacterial infection or invasive fungal disease (IFD) is suspected.

Procalcitonin (PCT) is a prohormone of calcitonin produced during systemic infection in response to circulating microbial toxins and host inflammatory mediators [2], [3]. Previous studies in non-neutropenic patients have reported the utility of PCT for differentiating bacterial from viral infections or non-infectious inflammatory conditions [2], [3], [4], [5], [6]. PCT concentrations thresholds have been proposed for distinguishing systemic from localized infections and infections due to virulent pathogens from those due to pathogens with low virulence (e.g. coagulase-negative staphylococci, CNS). Christ-Crain *et al* have shown that a PCT-based management algorithm reduces antibiotic use in lower respiratory tract infections [7], [8]. A high variability of the diagnostic performance of PCT has been reported in febrile neutropenic patients, in whom the utility of this blood marker remains unclear [9]. Few data are available on PCT in persistent neutropenic fever and suspected IFD. We aimed at prospectively monitoring the PCT kinetics in febrile neutropenic patients for the initial assessment of the etiology of fever and the diagnostic reassessment in persistent fever.

## Methods

### Patients

This study was conducted in the Isolation Ward of the Infectious Diseases Service of a 900-bed University Hospital during an 18-month period. Consecutive adult patients undergoing induction or consolidation myelosuppressive chemotherapy for acute leukemia, or intensive chemotherapy followed by autologous hematopoietic peripheral stem cell transplantation were enrolled after written informed-consent. All patients were hospitalized in single-bed isolation rooms with positive pressure and HEPA-filtered ventilation. The protocol was reviewed and accepted by the local HREC (Commission d'éthique de la recherche clinique, Faculté de Médecine et de Biologie, Université de Lausanne, approval number 179/01 on the 5th November 2001). All patients gave written informed consent prior to study enrolment.

### Febrile episodes

Neutropenia was defined as a neutrophil count <500/mm^3^. A febrile neutropenic episode (FE) was defined as an axillary temperature >38.5°C once or ≥38°C on two or more occasions (at least one hour apart) during a 12-h period [1]. A new febrile episode was defined as a relapsing fever after more than 72 hours of apyrexia (<38°C). For each FE the initial diagnostic work-up consisted of a thorough physical examination, two sets of aerobic and anaerobic blood cultures drawn by phlebotomy and via each line of the central venous catheter, urine culture, and chest X-ray. Additional specific investigations were performed according to the clinical presentation (e.g.: PCR for HSV on oropharyngeal swabs in patients with severe mucositis, screening of respiratory viruses with specific PCRs in patients with suggestive symptoms/signs). In persistent fever during more than 3 days, the diagnostic reassessment included repeated blood cultures as described above, 2-weekly measurement of galactomannan antigenemia (results were not available in real-time for clinical decisions, and only used for diagnostic classification of IFD: see next paragraph), a thoraco-abdominal CT-scan, a bronchoscopy with BAL in presence of a lung infiltrate, and biopsy for culture and histopathology at any clinically suspected site of infection.

### Classification of febrile episodes

Based on clinical, microbiological and radiological data and blinded of PCT results, the etiology of fever was classified on day 2 after onset of fever according to standard definitions of the International Immunocompromised Host Society (ICHS) and to the PCT manufacturer's recommendations derived from previous PCT studies: i) microbiologically documented infection with (MDIB) or without (MDInB) bacteremia [all MDI were differentiated in those due to coagulase-negative staphylococci (CNS) and those due to other pathogens (non-CNS)] [9], [10], [11], ii) clinically documented infection [CDI, subdivided into superficial (upper respiratory tract infections, mucositis grade 3, skin and soft tissue infections, lower urinary tract infections) and deep-seated infections (lower respiratory tract infections, enterocolitis, mucositis grade 4, hepato-splenic infection, arthritis, osteomyelitis, upper urinary tract infection, and CNS infection) ] [9], [12], [13], [14], iii) and fever of unexplained origin (FUO) [15]. Standard definitions of the focus/i of infection were used in CDI [16]. Mucositis with a WHO score >2 [17] and diarrhea with a frequency>8×/day were considered as CDI [18], [19]

Diagnosis of proven, probable or possible invasive fungal disease (IFD) was based on the definitions of the European Organization for the Research and Treatment of Cancer-Mycoses Study Group (EORTC-MSG) [20]. Galactomannan (Platelia, Biorad, France) and beta-glucan (colorimetric assay, Wako-Maruha, Japan [21]) antigenemias were measured on the same days as the PCT according to the recommendations of the manufacturers. IFD were included in the classification of infection as MDIB (fungemia), MDINB (tissue invasion documented by culture) or CDI (probable or possible IFD without microbiological evidence).

### Antimicrobial therapy

Broad-spectrum beta-lactam antibiotic monotherapy was started within 1 hour after onset of fever and adjusted after 48 hours according to microbiological results and clinical course, as recommended [1]. If a patient remained febrile during more than 3 days despite appropriate antibacterial therapy and/or presented signs of IFD, antifungal treatment was added as recommended [1]. Clinical, laboratory, radiological, microbiological and histopathological data and information on antimicrobial therapy were prospectively collected. Ongoing antimicrobial therapy was defined as inappropriate if a microbiologically documented (e.g. positive culture or PCR) or presumed (e.g. probable or possible IFD based on EORTC-MSG criteria) pathogen was not covered [22]. Antibacterial and antifungal prophylaxis were not used. Antiviral prophylaxis (valacyclovir) was administered to patients with positive HSV serology during the neutropenic period.

### Procalcitonin measurements

For PCT measurements, 5 ml–blood samples (Li-heparinate, Monovette®, Sarstedt, Numbrecht, Germany) were collected for each FE at the same time as blood cultures at onset of fever (day 0) and then on day 1, 2, 3, 4, and 7. Follow up after day 7 included twice-weekly blood sampling until occurrence of a subsequent FE (measurements according to the above sequence) or discharge (end of study). The same screening strategy was applied regardless of the resolution or the persistence of fever. Tubes were centrifuged during 10 minutes at 3000 rpm.min^−1^ and stored at −80°C until processed. PCT was measured by an amplified cryptate emission technology assay (Kryptor PCT, BRAHMS Diagnostica, Hennigsdorf bei Berlin, Germany) with a functional sensitivity of 60 pg/ml (corresponding to 0.06 ng/ml) and an analytical range of 60 pg/ml to 5000 pg/ml (i.e. 0.06 to 5 ng/ml). The test's intra- and inter-assay coefficient of variation was below 10% [23] Measurements of PCT were performed in batch at the end of study and were not available for patients management, nor for classification of the etiology of fever and localization of infection, as described above.

### Analyses of PCT results for initial diagnostic assessment of neutropenic fever

Daily PCT values over 7 days after onset of fever were compared among different etiologies of fever, as defined above. The time point with the highest PCT value (i.e. peak value) was considered for analyses of the performance of PCT cut-offs ranging 100–2000 pg/mL for distinguishing etiologies of fever in receiver operating characteristic (ROC) curves and by calculation of sensitivity, specificity, positive and negative predictive values and likelihood ratios, and efficiency. The best PCT cut-off was defined on the basis of the highest calculated efficiency.

### Analyses of PCT results for diagnostic reassessment in persistent neutropenic fever

PCT kinetics was analyzed in patients with persistent fever during more than 3 days focusing on the assessment of the appropriateness of ongoing antimicrobial therapy and the diagnosis of proven, probable or possible IFD.

### Statistical methods

Continuous variables were compared using the Mann-Whitney (2-group comparison) or Kruskall Wallis (multiple-group comparison) non-parametric tests. Categorical variables were compared with the χ^2^ or Fischer's exact test, as appropriate. A two-sided *p* value of less than 0.05 was considered as statistically significant. All statistical analyses were performed in Statistical Package for Social Science (SPSS® 15.0 for Windows, Chicago, Illinois, USA).

## Results

### Patients and febrile episodes

194 consecutive febrile episodes (FE) occurred during 125 neutropenic episodes in 90 hematological patients (median 1 FE per neutropenic episode, range 0–4). 1771 plasma samples were prospectively collected (median 13 per neutropenic episode, range 4–49). Patient demographics and characteristics of FE are shown in **Table 1**.

**Table 1:** Characteristics of patients and febrile episodes.

**Patients**	**90**
Median age (range)	57 (18–76)
Male/Female	55 (61%)/35 (39%)
Acute leukemia/Multiple myeloma or Lymphoma	49 (54%)/41 (46%)
**Neutropenic episodes**	**125**
Induction or consolidation for acute leukemia/Autologous HSCT	71 (57%)/54 (43%)
Median days of neutropenia (range)	15 (3–114)
Median days of hospitalization (range)	28 (15–122)
**Febrile episodes**	**194**
First/Recurrent febrile episode	133 (69%)/61 (31%)
Antibiotics/No antibiotics ongoing at onset of fever	85 (44%)/109 (56%)
**Microbiologically documented infection with bacteremia (MDIB)**	**45 (23%)**
Gram positive	24 (53%)
CNS/non – CNS^1^	12/12
Gram negative^2^	21 (47%)
**Microbiologically documented infection, non-bacteremic (MDInB)**	**20 (10.5%)**
Gram positive	9 (45%)
CNS/non – CNS^3^	4/5
Gram negative^4^	4 (20%)
Fungi	7 (35%)
**Clinically documented infections (CDI)**	**68 (35%)**
Superficial	29 (43%)
Mucositis grade 3	17
Soft tissue	10
Urinary tract infection	2
Deep-seated	39 (57%)
Pneumonia	17
Enterocolitis	11
Mucositis grade 4	8
Hepato-splenic candidiasis	3
**Fever of unknown origin (FUO)**	**61 (31.5%)**
**Severity of infection**	
Severe sepsis/septic shock	4 (2.0%)/0 (0%)
**Invasive fungal diseases (IFD)**	**25 (13%)**
Proven or probable	20 (80%)
Aspergillosis/Candidiasis	16/4
Possible	5 (20%)

1 *Streptococcus* spp (3), *Enterococcus* spp (3), other Gram positive (6).

2 *Escherichia coli* (10), *Klebsiella oxytoca* (2), *Klebsiella oxytoca* and *Citrobacter koseri* (3), *Enterobacter cloacae* (1), *Enterobacter cloacae* and *Moraxella* catharralis (1), other Gram negative (4).

3 *Clostridium difficile* (5).

4 *Klebsiella pneumoniae* (2), *Escherichia coli* (1), *Pseudomonas aeruginosa* (1).

HSCT = hematopoietic stem cell transplantation.

CNS = coagulase-negative staphylococci.

### PCT for initial diagnostic assessment of neutropenic fever

**Figure 1:**
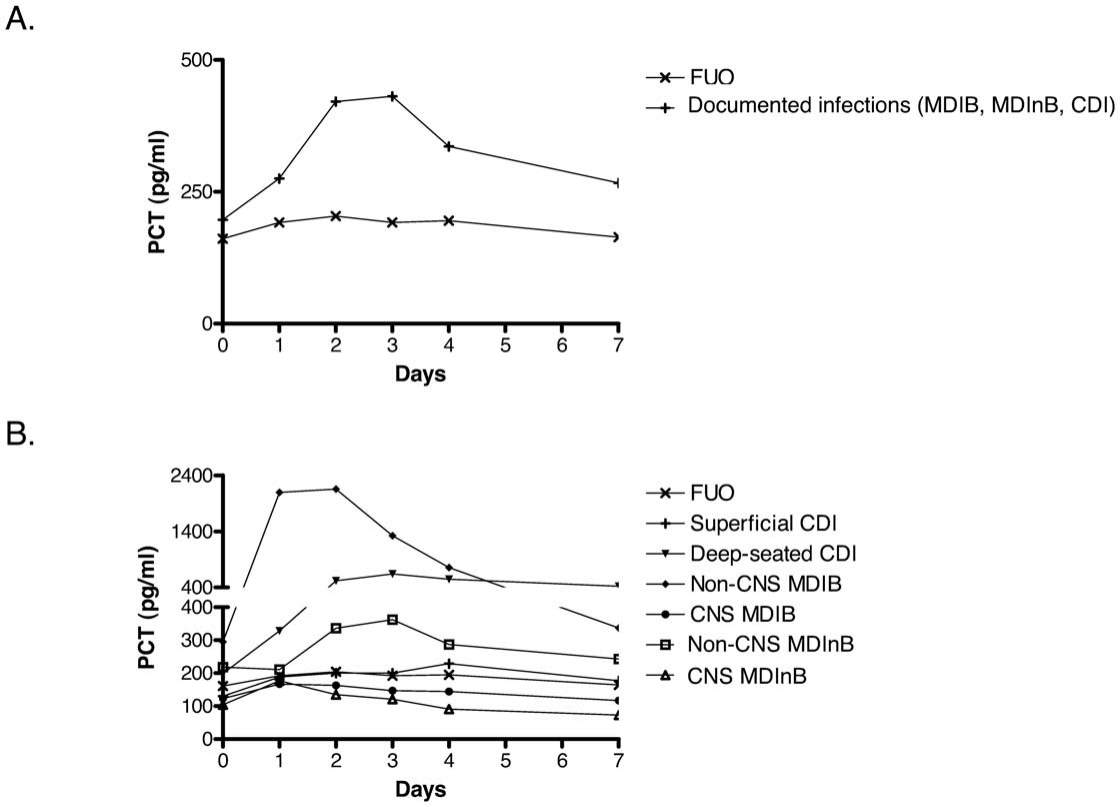
Median PCT values over 7 days after onset of fever according to the etiology of fever and the localization of infection. 1A: documented infections (MDIB, MDInB, and CDI) and FUO. 1B: non-CNS MDIB, non-CNS MDInB, CNS MDIB, CNS MDInB, superficial CDI, deep-seated CDI, and FUO. Day 0 = onset of fever, MDIB = microbiologically documented infections with bacteremia, MDInB = microbiologically documented infections without bacteremia, CDI = clinically documented infections, FUO = fever of unknown origin, CNS = coagulase-negative staphylococci.

The PCT kinetics over 7 days in FE of different etiologies is shown in **Figure 1** **, Panel A and B**. At fever onset (day 0) no significant difference in PCT values was observed between documented infections (MDIB, MDInB, CDI) and FUO. When subgroups were analyzed, PCT values were significantly higher in non-CNS MDIB (median 295 pg/mL, range 55–26800) when compared with all the other etiologies of fever (median 161 pg/mL, range 30–1751; p<0.05). Among MDIB, PCT at fever onset was statistically superior in Gram-negative when compared to Gram-positive bacteremia (median 321 [range 54–14660] vs 129 [57–26800], p = 0.004). However, if CNS-MDIB were removed from this comparison, no statistically significant difference was observed (median 321 [range 54–14660] vs 150 [73–26800], p = 0.183).

**Figure 2:**
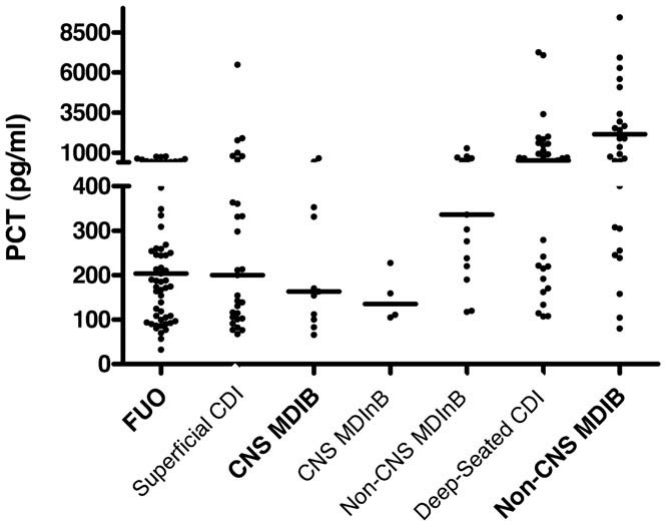
Individual PCT values on day 2 after onset of fever according to etiology of fever and localization of infection. Each point represents the PCT value in a single febrile episode. Horizontal bars represent median values in each group. MDIB = microbiologically documented infections with bacteremia, MDInB = microbiologically documented infections without bacteremia, CDI = clinically documented infections, FUO = fever of unknown origin, CNS = coagulase-negative staphylococci.

The PCT peak was observed at a median of 2 days after the onset of fever (range 0–20). When compared with PCT values on day 2 in FUO (median 204, range 33–771), those in non-CNS MDIB (2155 pg/ml, range 80–86350; p<0.001), non-CNS MDInB (336, range 117–12090; p = 0.007) and deep-seated CDI (median 520, range 107–13000; p<0.001) were significantly higher. However, no significant difference in PCT values on day 2 was observed in CNS MDIB/MDInB and superficial CDI when compared with FUO (**Figure 2**). No difference in PCT values in the various etiologies of fever was observed by stratifying for the type of chemotherapy regimen, i.e. induction/consolidation chemotherapy for acute leukemia vs. conditioning chemotherapy followed by autologous hematopoietic stem cell transplantation (data not shown).

**Figure 3:**
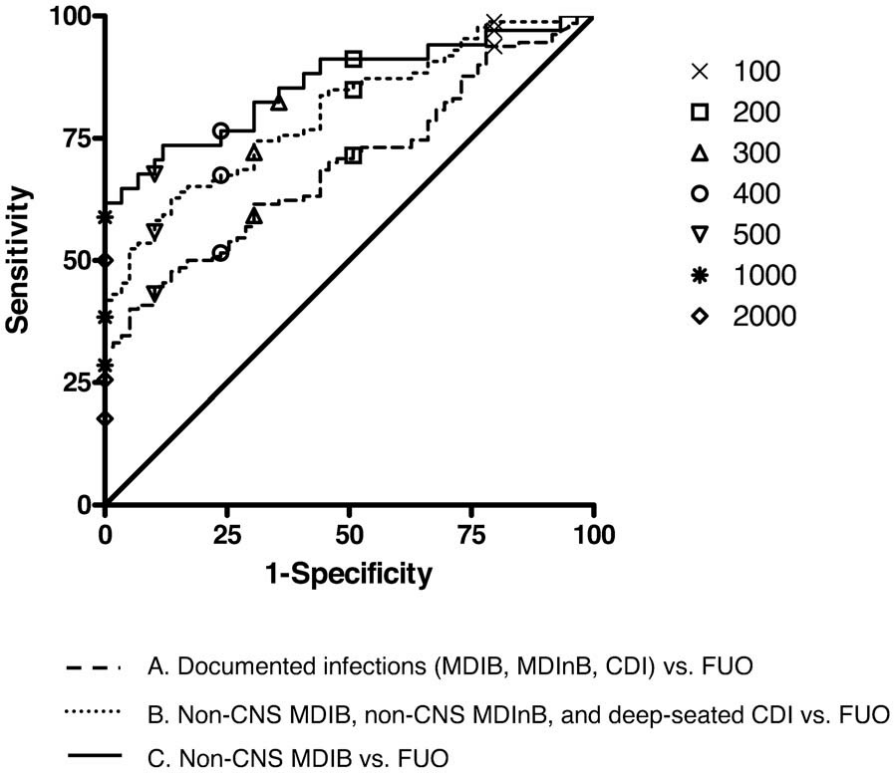
Receiver operating characteristic curve of PCT on day 2 after onset of fever for diagnosis of A. documented infections (MDIB, MDInB, CDI) vs. FUO (AUC = 0.695); B. non-CNS MDIB, non-CNS MDInB, and deep-seated CDI vs. FUO (AUC = 0.803); and C. non-CNS MDIB vs. FUO (AUC = 0.860). MDIB = microbiologically documented infections with bacteremia, MDInB = microbiologically documented infections without bacteremia, CDI = clinically documented infections, FUO = fever of unknown origin, CNS = coagulase-negative staphylococci, AUC = area under the receiver operating characteristic curves.

PCT cut-offs ranging between 100 and 2000 pg/mL were analyzed on day 2 after the onset of fever. Proportions of PCT values higher than above cut-offs in different FE etiologies are reported in **Table 2**. The performances of these PCT cut-offs for discriminating different subsets of documented infections from FUO were calculated. The areas under the ROC curves were 0.695 (all documented infections vs. FUO), 0.803 (non-CNS MDI and deep-seated CDI vs. FUO), and 0.860 (non-CNS MDIB vs. FUO), respectively (**Figure 3**). The best diagnostic efficiency was observed with a PCT cut-off of 500 pg/mL (**Table 3**). Efficiency of PCT was not different in first vs. recurrent FE, in those occurring with and without ongoing antibiotic therapy at time of onset of fever (data not shown).

**Table 2:** PCT on day 2 after onset of fever: positivity at different cut-offs in i) all FE, ii) documented infections (MDIB, MDInB and CDI), iii) subset including non-CNS MDIB, non-CNS MDInB, and deep-seated CDI, iv) non-CNS MDIB, and v) FUO.

PCT cut-off (pg/mL)	Febrile episodes	Documented infections (MDI and CDI)	Non-CNS MDIB, non-CNS MDInB and deep-seated CDI	Non-CNS MDIB	FUO
	n = 189*	n = 130	n = 86	n = 33	n = 59
**≥100**	169 (88.4%)	122 (93.8%)	85 (98.8%)	33 (100%)	47 (79.7%)
**≥200**	123 (65.1%)	93 (71.5%)	73 (84.9%)	31 (93.9%)	30 (50.8%)
**≥300**	95 (50.3%)	77 (59.2%)	62 (72.1%)	28 (84.8%)	18 (30.5%)
**≥400**	81 (42.9%)	67 (51.5%)	58 (67.5%)	26 (78.8%)	14 (23.7%)
**≥500**	62 (32.8%)	56 (43.1%)	48 (55.8%)	23 (69.7%)	6 (10.2%)
**≥1000**	37 (19.6%)	37 (28.5%)	33 (38.4%)	20 (60.6%)	0 (0%)
**≥2000**	23 (12.2%)	23 (17.7%)	22 (25.6%)	17 (51.5%)	0 (0%)

*In 5 febrile episodes (3 documented infections and 2 FUO), blood sampling on day 2 was missing.

MDIB = microbiologically documented infections with bacteremia, MDInB = microbiologically documented infections without bacteremia, CDI = clinically documented infections, FUO = fever of unknown origin, CNS = coagulase-negative staphylococci.

**Table 3:** Diagnostic performance of PCT at the 500 pg/mL cut-off on day 2 after onset of fever in i) documented infections (MDIB, MDInB and CDI) vs. FUO, ii) non-CNS MDIB, non-CNS MDInB and deep-seated CDI vs. FUO, and iii) non-CNS MDIB vs. FUO.

Etiology of fever	Sensitivity (95% CI)	Specificity (95% CI)	PPV (95% CI)	NPV (95% CI)	Positive LR (95% CI)	Negative LR (95% CI)	Efficiency
**Documented infections vs. FUO**	43.1% (34.5–52.0)	89.8% (78.5–95.8)	90.3% (79.5–96.0)	41.7% (33.2–50.8)	4.26 (1.93–9.27)	0.63 (0.54–0.74)	0.58
**Non-CNS MDIB/MDInB and deep-seated CDI vs. FUO**	55.8% (44.7–66.4)	89.8% (78.5–95.8)	88.9% (76.7–95.4)	58.2% (47.4–68.3)	5.49 (2.51–12.00)	0.49 (0.39–0.63)	0.70
**Non-CNS MDIB vs. FUO**	69.7% (51.1–83.8)	89.8% (78.5–95.8)	79.3% (59.7–91.3)	84.1% (72.3–91.7)	6.85 (3.11–15.12)	0.34 (0.20–0.57)	0.83

PPV = positive predictive value, NPV = negative predictive value, LR = likelihood ratio. 95%CI = 95% confidence interval.

MDIB = microbiologically documented infections with bacteremia, MDInB = microbiologically documented infections without bacteremia, CDI = clinically documented infections, FUO = fever of unknown origin, CNS = coagulase-negative staphylococci.

On day 2, 6/59 (10.2%) FUO had a PCT higher than 500 pg/mL (median 688, range 570–771): these PCT values were comparable to those observed in deep-seated CDI. FUO with positive or negative PCT did not differ significantly for maximal temperature (median 39°C, range 38–39.6, vs. 38.6, 38–40.1) or duration of fever (median 1.5 day, range 1–12, vs. 1, 1–15). All patients responded to antibiotic therapy. Interestingly, although PCT results were not disclosed in real-time for clinical decisions, the duration of therapy was longer in FUO with positive vs. in those with negative PCT (median 9.5 days, range 5–27, vs. 6, 0–25, p = 0.013),

### PCT for diagnostic reassessment in persistent neutropenic fever

Fever persisted during more than 3 days in 63/194 (32%) FE (20 MDI, 31 CDI, 12 FUO). Antimicrobial therapy was inappropriate in 24/63 (38%) cases (18 invasive fungal diseases [IFD], 5 bacterial and 1 viral infections due to VZV reactivation). The proportion of persistently febrile episodes with inappropriate antimicrobial therapy was significantly higher in those with a PCT higher than 500 pg/mL (13/24, 54%) when compared with those with a PCT lower than 500 pg/mL (11/39, 28%; p = 0.039).

**Figure 4:**
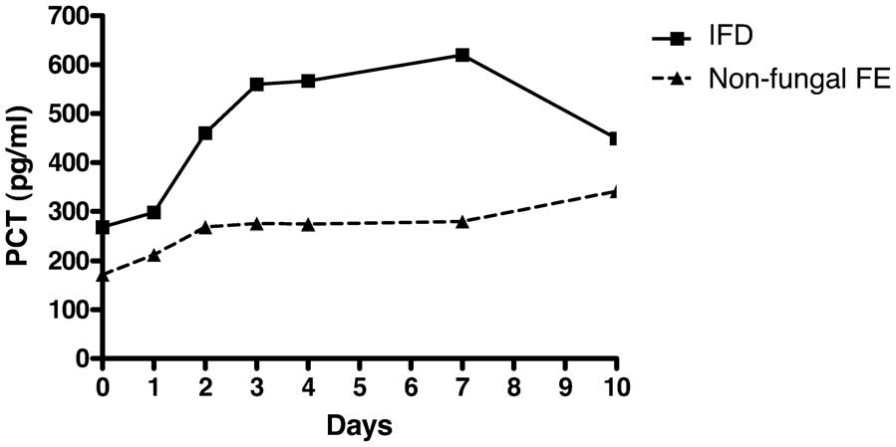
PCT kinetics in persistent neutropenic fever during more than 3 days: IFD vs. non-fungal FE. Median PCT values at each time point are shown.

IFD according to EORTC-MSG criteria were diagnosed in 21/63 (33%) episodes of persistent fever (18 invasive aspergillosis: 1 proven, 12 probable, 5 possible; 3 invasive candidiasis: 1 proven, 2 probable). **Figure 4** shows the PCT kinetics in persistent fever beyond day 3 comparing IFD (median PCT 560 pg/ml, range 32–11950) with non-fungal FE (median PCT 299 pg/ml, range 0–166200 [p = 0.001 vs. IFD]). A delayed PCT peak (increase >500 pg/ml beyond 3 days after fever onset) was observed in 17/21 (81%, median 1196 pg/ml, range 524–11950) IFD with persistent fever when compared with 18/42 (43%) non-fungal FE with persistent fever (p = 0.004). The performance of a delayed PCT peak for the diagnosis of proven, probable or possible IFD in persistent fever during more than 3 days was: sensitivity 81% (95%CI 57–94%), specificity 57% (95%CI 41–72%), PPV 49% (95%CI 32–66%), NPV 86% (95%CI 66–95%), positive likelihood ratio 1.89 (95%CI 1.26–2.84), negative likelihood ratio 0.33 (95%CI 0.13–0.83%), efficiency 0.65. The diagnostic performance of PCT for proven and probable IFD only (after exclusion of the 5 possible cases from the analysis) was similar (data not shown). The delayed PCT increase above 500 pg/mL in IFD with persistent fever occurred in the same time window as the diagnosis of IFD according to EORTC-MSG criteria: median 7 days after onset of fever, range 0–21, vs. 8 days, range 4–20 (NS). Delayed PCT increase above 500 pg/mL preceded diagnosis based on EORTC-MSG criteria in 4/17 (24%) IFD cases and was simultaneous in 4/17 (24%).

**Figure 5:**
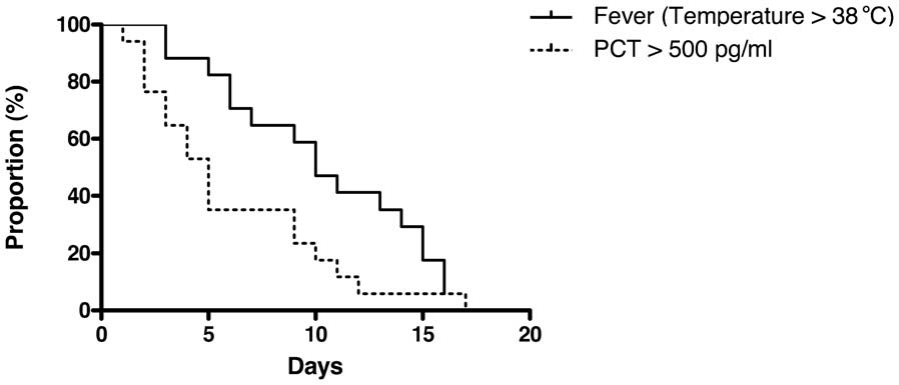
Percent of patients with PCT >500 pg/ml and fever (temperature >38°C) in follow-up of IFD. The T0 for PCT and fever is set on the time point at which the first positive PCT value (>500 pg/ml) has been documented.

The patients with IFD received an antifungal therapy within a median of 4 days (range 0–10) after onset of fever: all survived and were afebrile at time of discharge. The proportions of patients with PCT higher than 500 pg/mL and with fever during the course of IFD are shown in **Figure 5**. The PCT decrease below 500 pg/mL preceded the resolution of fever: median 5 days (range 1–23) vs. 10 days (range 3–22), respectively (p = 0.026).

## Discussion

The kinetics of PCT was prospectively studied in a large cohort of febrile neutropenic patients with haematological malignancies at high risk for severe bacterial and fungal infections. A close monitoring of this blood marker at different time points during the course of a febrile episode and a thorough analysis of a broad range of PCT cut-offs provided reliable information on its utility for the initial assessment of the etiology of fever and for the diagnostic reassessment in persistent fever.

At onset of fever, PCT was low to undetectable (i.e. below the threshold of positivity), although the values in non-CNS MDIB were significantly higher than those in all other etiologies of fever. A PCT peak was observed in non-CNS MDI and deep-seated CDI 2 days after the onset of fever, i.e. the time at which a re-evaluation of the empirical antibacterial therapy based on clinical course and microbiological results is recommended. The PCT threshold of 500 pg/mL allowed the best discrimination of these severe infections from infections due to CNS, superficial infections or FUO. Interestingly, PCT values higher than this threshold were observed in a minority of patients with FUO, suggesting that these FE may have been due to deep-seated bacterial infections with lacking clinical and microbiological documentation. However, this could not be demonstrated in these few patients in whom fever was the only clinical sign suggesting infection and who had received empirical antibacterial therapy with prompt defervescence.

Monitoring PCT for the diagnostic reassessment in patients with persistent neutropenic fever lasting more than 3 days revealed that a delayed PCT peak higher than 500 pg/mL was more frequently observed in patients receiving inappropriate antimicrobial therapy. This finding was true in the majority of cases of IFD and often preceded diagnosis of IFD based on EORTC-MSG criteria. In follow-up PCT decline reflecting the response of IFD to antifungal therapy significantly anticipated defervescence.

Previous investigations in febrile neutropenic patients have reported controversial results regarding the performance of PCT for the initial assessment of the etiology of fever by distinguishing bacteremia due to pathogens other than CNS from non-bacteremic infections, and systemic from localized infections or FUO [9], [10], [11], [12], [24], [25], [26], [27], [28], [29], [30].In the present study, the low PCT values observed at the time of onset of fever in severe documented infections do not support the use of PCT for the decision to start or withhold prompt empirical antibacterial therapy. This finding differs from previous reports in which the timing of the first PCT measurement was variable [9], [12], [26], [28], [29], i.e. it corresponded either to the onset of fever, the onset of antimicrobial therapy [11] or the time of blood cultures [10]. Moreover, different types of neutropenic populations (i.e. hematological vs solid tumors, adults vs children) and delayed PCT measurements in outpatients vs inpatients may be responsible of the heterogeneity of the results in some studies. On the other hand, the lack of a reliable diagnostic tool for distinguishing infections from non-infectious causes of fever when microbiological and clinical documentation is lacking, i.e. in FUO, remains a major issue in management of febrile neutropenic patients. The utility of PCT for identifying non-documented bacterial infections in patients with FUO has been suggested by Giamarellos-Bourboulis *et al.* who reported higher PCT values in those responding to antibacterial therapy when compared with those without response [12]. In the present study, PCT values similar to those observed in deep-seated CDI were measured on day 2 after onset of fever in a small subset of patients with FUO, which might help to identify febrile episodes due to occult severe bacterial infections.

New findings are provided on the role of PCT for the diagnostic reassessment in patients with persistent neutropenic fever and suspected IFD. Few data have been reported in this specific setting, which represents a major challenge for the clinical management. Small case series have described controversial results on the utility of PCT for the diagnosis of IFD [31], [32], [33], [34]. Some did not report any increase of PCT concentrations in life-threatening IFD [31], [32], [33], while others associated elevated PCT values with poor outcome in IFD [34]. In 5 cases of invasive aspergillosis in allogeneic hematopoietic stem cell transplant recipients, PCT values exceeding 3000 pg/mL have been described [35], [36]. In contrast with the paucity of available data in the literature, sequential PCT measurements in a large number of episodes of persistent fever (n = 63), including the highest number of IFD reported in a PCT study (n = 25) are analyzed in the present investigation. The results suggest that a delayed PCT peak higher than 500 pg/mL observed beyond 3 days of persistent fever is helpful for the early diagnosis of IFD. This blood marker could be combined with the conventional radiological and microbiological tools for improving and anticipating the detection of these life-threatening infections. Moreover, a PCT decrease to normal levels significantly preceded the resolution of fever in follow-up of IFD responding to antifungal therapy. Monitoring PCT may thus help to avoid unnecessary investigations and modifications of antimicrobial therapy in IFD with prolonged fever.

In contrast with previous studies, which have shown that persistently elevated or increasing PCT values predict the outcome in neutropenic patients with severe sepsis or septic shock, a very low rate of severe septic complications was observed in the present investigation. Prompt and appropriate empirical antibacterial therapy in an epidemiological setting of low bacterial resistance has probably influenced the PCT kinetics in the majority of potentially life-threatening infections, thus precluding the evaluation of PCT as a predictor of infection-related severe morbidity and mortality [9], [12], [37], [38], [39]. The lack of data in allogeneic hematopoietic transplant recipients and the small number of proven IFD are other limitations of this single-center study.

In conclusion, when recommended clinical, radiological and microbiological investigations are used, the utility of PCT for the initial assessment of the etiology of febrile neutropenia is limited. While PCT measured at the onset of fever cannot be used for the decision to start or withhold immediate empirical antibacterial therapy, a PCT value higher than 500 pg/mL in FUO on day 2 might suggest an occult bacterial infection, i.e. with lacking microbiological and clinical documentation. In contrast, PCT monitoring is useful for diagnostic reassessment in persistent fever. A delayed PCT peak higher than 500 pg/mL contributes to the early diagnosis of invasive fungal disease and PCT decrease in follow-up promptly reflects response to antifungal therapy when compared with the slow resolution of fever.

## References

[1] Hughes WT, Armstrong D, Bodey GP, Bow EJ, Brown AE (2002). 2002 guidelines for the use of antimicrobial agents in neutropenic patients with cancer.. Clin Infect Dis.

[2] Scire CA, Cavagna L, Perotti C, Bruschi E, Caporali R (2006). Diagnostic value of procalcitonin measurement in febrile patients with systemic autoimmune diseases.. Clin Exp Rheumatol.

[3] Muller B, Becker KL, Schachinger H, Rickenbacher PR, Huber PR (2000). Calcitonin precursors are reliable markers of sepsis in a medical intensive care unit.. Crit Care Med.

[4] Kuse ER, Langefeld I, Jaeger K, Kulpmann WR (2000). Procalcitonin in fever of unknown origin after liver transplantation: a variable to differentiate acute rejection from infection.. Crit Care Med.

[5] Viallon A, Zeni F, Lambert C, Pozzetto B, Tardy B (1999). High sensitivity and specificity of serum procalcitonin levels in adults with bacterial meningitis.. Clin Infect Dis.

[6] Gilbert DN (2010). Use of plasma procalcitonin levels as an adjunct to clinical microbiology.. J Clin Microbiol.

[7] Christ-Crain M, Jaccard-Stolz D, Bingisser R, Gencay MM, Huber PR (2004). Effect of procalcitonin-guided treatment on antibiotic use and outcome in lower respiratory tract infections: cluster-randomised, single-blinded intervention trial.. Lancet.

[8] Christ-Crain M, Stolz D, Bingisser R, Muller C, Miedinger D (2006). Procalcitonin guidance of antibiotic therapy in community-acquired pneumonia: a randomized trial.. Am J Respir Crit Care Med.

[9] Giamarellou H, Giamarellos-Bourboulis EJ, Repoussis P, Galani L, Anagnostopoulos N (2004). Potential use of procalcitonin as a diagnostic criterion in febrile neutropenia: experience from a multicentre study.. Clin Microbiol Infect.

[10] Persson L, Engervall P, Magnuson A, Vikerfors T, Soderquist B (2004). Use of inflammatory markers for early detection of bacteraemia in patients with febrile neutropenia.. Scand J Infect Dis.

[11] Ruokonen E, Nousiainen T, Pulkki K, Takala J (1999). Procalcitonin concentrations in patients with neutropenic fever.. Eur J Clin Microbiol Infect Dis.

[12] Giamarellos-Bourboulis EJ, Grecka P, Poulakou G, Anargyrou K, Katsilambros N (2001). Assessment of procalcitonin as a diagnostic marker of underlying infection in patients with febrile neutropenia.. Clin Infect Dis.

[13] Pulliam PN, Attia MW, Cronan KM (2001). C-reactive protein in febrile children 1 to 36 months of age with clinically undetectable serious bacterial infection.. Pediatrics.

[14] Andreola B, Bressan S, Callegaro S, Liverani A, Plebani M (2007). Procalcitonin and C-reactive protein as diagnostic markers of severe bacterial infections in febrile infants and children in the emergency department.. Pediatr Infect Dis J.

[15] The Immunocompromised Host Society (1990). The design, analysis, and reporting of clinical trials on the empirical antibiotic management of the neutropenic patient. Report of a consensus panel.. J Infect Dis.

[16] JS G, WR J, TG E, TC H, JM H, Practice AICaAEPa, editor (1996). CDC definitions for nosocomial infections;.

[17] Blijlevens N, Schwenkglenks M, Bacon P, D'Addio A, Einsele H (2008). Prospective oral mucositis audit: oral mucositis in patients receiving high-dose melphalan or BEAM conditioning chemotherapy–European Blood and Marrow Transplantation Mucositis Advisory Group.. J Clin Oncol.

[18] Kern WV, Cometta A, De Bock R, Langenaeken J, Paesmans M (1999). Oral versus intravenous empirical antimicrobial therapy for fever in patients with granulocytopenia who are receiving cancer chemotherapy. International Antimicrobial Therapy Cooperative Group of the European Organization for Research and Treatment of Cancer.. N Engl J Med.

[19] Cometta A, Kern WV, De Bock R, Paesmans M, Vandenbergh M (2003). Vancomycin versus placebo for treating persistent fever in patients with neutropenic cancer receiving piperacillin-tazobactam monotherapy.. Clin Infect Dis.

[20] De Pauw B, Walsh TJ, Donnelly JP, Stevens DA, Edwards JE (2008). Revised definitions of invasive fungal disease from the European Organization for Research and Treatment of Cancer/Invasive Fungal Infections Cooperative Group and the National Institute of Allergy and Infectious Diseases Mycoses Study Group (EORTC/MSG) Consensus Group.. Clin Infect Dis.

[21] Senn L, Robinson JO, Schmidt S, Knaup M, Asahi N (2008). 1,3-Beta-D-glucan antigenemia for early diagnosis of invasive fungal infections in neutropenic patients with acute leukemia.. Clin Infect Dis.

[22] Institute. CaLSInstitute. CaLS, editor (2006). Methods for Dilution Antimicrobial Susceptibility Tests for Bacteria that Grow Aerobically—Seventh Edition: Approved Standard M7-A7.;.

[23] Steinbach G, Rau B, Debard AL, Javourez JF, Bienvenu J (2004). Multicenter evaluation of a new immunoassay for procalcitonin measurement on the Kryptor System.. Clin Chem Lab Med.

[24] Sakr Y, Sponholz C, Tuche F, Brunkhorst F, Reinhart K (2008). The role of procalcitonin in febrile neutropenic patients: review of the literature.. Infection.

[25] Engel A, Steinbach G, Kern P, Kern WV (1999). Diagnostic value of procalcitonin serum levels in neutropenic patients with fever: comparison with interleukin-8.. Scand J Infect Dis.

[26] Fleischhack G, Kambeck I, Cipic D, Hasan C, Bode U (2000). Procalcitonin in paediatric cancer patients: its diagnostic relevance is superior to that of C-reactive protein, interleukin 6, interleukin 8, soluble interleukin 2 receptor and soluble tumour necrosis factor receptor II.. Br J Haematol.

[27] Jimeno A, Garcia-Velasco A, del Val O, Gonzalez-Billalabeitia E, Hernando S (2004). Assessment of procalcitonin as a diagnostic and prognostic marker in patients with solid tumors and febrile neutropenia.. Cancer.

[28] Schuttrumpf S, Binder L, Hagemann T, Berkovic D, Trumper L (2006). Utility of procalcitonin concentration in the evaluation of patients with malignant diseases and elevated C-reactive protein plasma concentrations.. Clin Infect Dis.

[29] von Lilienfeld-Toal M, Dietrich MP, Glasmacher A, Lehmann L, Breig P (2004). Markers of bacteremia in febrile neutropenic patients with hematological malignancies: procalcitonin and IL-6 are more reliable than C-reactive protein.. Eur J Clin Microbiol Infect Dis.

[30] Stryjewski GR, Nylen ES, Bell MJ, Snider RH, Becker KL (2005). Interleukin-6, interleukin-8, and a rapid and sensitive assay for calcitonin precursors for the determination of bacterial sepsis in febrile neutropenic children.. Pediatr Crit Care Med.

[31] Beaune G, Bienvenu F, Pondarre C, Monneret G, Bienvenu J (1998). Serum procalcitonin rise is only slight in two cases of disseminated aspergillosis.. Infection.

[32] Huber W, Schweigart U, Bottermann P (1997). Failure of PCT to indicate severe fungal infection in two immunodeficient patients.. Infection.

[33] Petrikkos GL, Christofilopoulou SA, Tentolouris NK, Charvalos EA, Kosmidis CJ (2005). Value of measuring serum procalcitonin, C-reactive protein, and mannan antigens to distinguish fungal from bacterial infections.. Eur J Clin Microbiol Infect Dis.

[34] Christofilopoulou S, Charvalos E, Petrikkos G (2002). Could procalcitonin be a predictive biological marker in systemic fungal infections? Study of 14 cases.. Eur J Intern Med.

[35] Ortega M, Rovira M, Filella X, Almela M, Puig de la Bellacasa J (2004). Prospective evaluation of procalcitonin in adults with febrile neutropenia after haematopoietic stem cell transplantation.. Br J Haematol.

[36] Ortega M, Rovira M, Filella X, Martinez JA, Almela M (2006). Prospective evaluation of procalcitonin in adults with non-neutropenic fever after allogeneic hematopoietic stem cell transplantation.. Bone Marrow Transplant.

[37] Brunkhorst FM, Wegscheider K, Forycki ZF, Brunkhorst R (2000). Procalcitonin for early diagnosis and differentiation of SIRS, sepsis, severe sepsis, and septic shock.. Intensive Care Med.

[38] Sauer M, Tiede K, Fuchs D, Gruhn B, Berger D (2003). Procalcitonin, C-reactive protein, and endotoxin after bone marrow transplantation: identification of children at high risk of morbidity and mortality from sepsis.. Bone Marrow Transplant.

[39] Svaldi M, Hirber J, Lanthaler AI, Mayr O, Faes S (2001). Procalcitonin-reduced sensitivity and specificity in heavily leucopenic and immunosuppressed patients.. Br J Haematol.

